# Efficacy and Brain Imaging Correlates of an Immersive Motor Imagery BCI-Driven VR System for Upper Limb Motor Rehabilitation: A Clinical Case Report

**DOI:** 10.3389/fnhum.2019.00244

**Published:** 2019-07-11

**Authors:** Athanasios Vourvopoulos, Carolina Jorge, Rodolfo Abreu, Patrícia Figueiredo, Jean-Claude Fernandes, Sergi Bermúdez i Badia

**Affiliations:** ^1^Neural Plasticity and Neurorehabilitation Laboratory, University of Southern California, Los Angeles, CA, United States; ^2^Madeira Interactive Technologies Institute, Universidade da Madeira, Funchal, Portugal; ^3^Institute for Systems and Robotics, Department of Bioengineering, Instituto Superior Técnico, Universidade de Lisboa, Lisbon, Portugal; ^4^Central Hospital of Funchal, Physical Medicine and Rehabilitation Service, Funchal, Portugal; ^5^Faculdade de Ciências Exatas e da Engenharia, Universidade da Madeira, Funchal, Portugal

**Keywords:** brain-computer interface, EEG, fMRI, virtual-reality, neurorehabilitation

## Abstract

To maximize brain plasticity after stroke, a plethora of rehabilitation strategies have been explored. These include the use of intensive motor training, motor-imagery (MI), and action-observation (AO). Growing evidence of the positive impact of virtual reality (VR) techniques on recovery following stroke has been shown. However, most VR tools are designed to exploit active movement, and hence patients with low level of motor control cannot fully benefit from them. Consequently, the idea of directly training the central nervous system has been promoted by utilizing MI with electroencephalography (EEG)-based brain-computer interfaces (BCIs). To date, detailed information on which VR strategies lead to successful functional recovery is still largely missing and very little is known on how to optimally integrate EEG-based BCIs and VR paradigms for stroke rehabilitation. The purpose of this study was to examine the efficacy of an EEG-based BCI-VR system using a MI paradigm for post-stroke upper limb rehabilitation on functional assessments, and related changes in MI ability and brain imaging. To achieve this, a 60 years old male chronic stroke patient was recruited. The patient underwent a 3-week intervention in a clinical environment, resulting in 10 BCI-VR training sessions. The patient was assessed before and after intervention, as well as on a one-month follow-up, in terms of clinical scales and brain imaging using functional MRI (fMRI). Consistent with prior research, we found important improvements in upper extremity scores (Fugl-Meyer) and identified increases in brain activation measured by fMRI that suggest neuroplastic changes in brain motor networks. This study expands on the current body of evidence, as more data are needed on the effect of this type of interventions not only on functional improvement but also on the effect of the intervention on plasticity through brain imaging.

## Introduction

Worldwide, stroke is a leading cause of adult long-term disability (Mozaffarian et al., 2015). From those who survive, an increased number is suffering with severe cognitive and motor impairments, resulting in loss of independence in their daily life such as self-care tasks and participation in social activities (Miller et al., 2010). Rehabilitation following stroke is a multidisciplinary approach to disability which focuses on recovery of independence. There is increasing evidence that chronic stoke patients maintain brain plasticity, meaning that there is still potential for additional recovery (Page et al., 2004). Traditional motor rehabilitation is applied through physical therapy and/or occupational therapy. Current approaches of motor rehabilitation include functional training, strengthening exercises, and range of movement exercises. In addition, techniques based on postural control, stages of motor learning, and movement patterns have been proposed such as in the Bobath concept and Bunnstrom approach (amongst others) (Bobath, 1990). After patients complete subacute rehabilitation programs, many still show significant upper limb motor impairment. This has important functional implications that ultimately reduce their quality of life. Therefore, alternative methods to maximize brain plasticity after stroke need to be developed.

So far, there is growing evidence that action observation (AO) (Celnik et al., 2008) and motor imagery (MI) improve motor function (Mizuguchi and Kanosue, 2017) but techniques based on this paradigm are not widespread in clinical settings. As motor recovery is a learning process, the potential of MI as a training paradigm relies on the availability of an efficient feedback system. To date, a number of studies have demonstrated the positive impact of virtual-reality (VR) based on neuroscientific grounds on recovery, with proven effectiveness in the stroke population (Bermúdez i Badia et al., 2016). However, patients with no active movement cannot benefit from current VR tools due to low range of motion, pain, fatigue, etc. (Trompetto et al., 2014). Consequently, the idea of directly training the central nervous system was promoted by establishing an alternative pathway between the user’s brain and a computer system.

This is possible by using electroencephalography (EEG)-based Brain-Computer Interfaces (BCIs), since they can provide an alternative non-muscular channel for communication and control to the external world (Wolpaw et al., 2002), while they could also provide a cost-effective solution for training (Vourvopoulos and Bermúdez, 2016b). In rehabilitation, BCIs could offer a unique tool for rehabilitation since they can stimulate neural networks through the activation of mirror neurons (Rizzolatti and Craighero, 2004) by means of action-observation (Kim et al., 2016), motor-intent and motor-imagery (Neuper et al., 2009), that could potentially lead to post-stroke motor recovery. Thus, BCIs could provide a backdoor to the activation of motor neural circuits that are not stimulated through traditional rehabilitation techniques.

In EEG-based BCI systems for motor rehabilitation, Alpha (8–12 Hz) and Beta (12–30 Hz) EEG rhythms are utilized since they are related to motor planning and execution (McFarland et al., 2000). During a motor attempt or motor imagery, the temporal pattern of the Alpha rhythms desynchronizes. This rhythm is also named Rolandic Mu-rhythm or the sensorimotor rhythm (SMR) because of its localization over the sensorimotor cortices. Mu-rhythms are considered indirect indications of functioning of the mirror neuron system and general sensorimotor activity (Kropotov, 2016). These are often detected together with Beta rhythm changes in the form of an event-related desynchronization (ERD) when a motor action is executed (Pfurtscheller and Lopes da Silva, 1999). These EEG patterns are primarily detected during task-based EEG (e.g., when the participant is actively moving or imagining movement) and they are of high importance in MI-BCIs for motor rehabilitation.

A meta-analysis of nine studies (combined *N* = 235, sample size variation 14 to 47) evaluated the clinical effectiveness of BCI-based rehabilitation of patients with post-stroke hemiparesis/hemiplegia and concluded that BCI technology could be effective compared to conventional treatment (Cervera et al., 2018). This included ischemic and hemorrhagic stroke in both subacute and chronic stages of stoke, between 2 to 8 weeks. Moreover, there is evidence that BCI-based rehabilitation promotes long-lasting improvements in motor function of chronic stroke patients with severe paresis (Ramos-Murguialday et al., 2019), while overall BCI’s are starting to prove their efficacy as rehabilitative technologies in patients with severe motor impairments (Chaudhary et al., 2016).

The feedback modalities used for BCI motor rehabilitation include: non-embodied simple two-dimensional tariffs on a screen (Prasad et al., 2010; Mihara et al., 2013), embodied avatar representation of the patient on a screen or with augmented reality (Holper et al., 2010; Pichiorri et al., 2015), neuromuscular electrical stimulation (NMES) (Kim et al., 2016; Biasiucci et al., 2018). and robotic exoskeletal orthotic movement facilitation (Ramos-Murguialday et al., 2013; Várkuti et al., 2013; Ang et al., 2015). In addition, it has been shown that multimodal feedback lead to a significantly better performance in motor-imagery (Sollfrank et al., 2016) but also multimodal feedback combined with motor-priming, (Vourvopoulos and Bermúdez, 2016a). However, there is no evidence which modalities are more efficient in stroke rehabilitation are.

Taking into account all previous findings in the effects of multimodal feedback in MI training, the purpose of this case study is to examine the effect of the MI paradigm as a treatment for post-stroke upper limb motor dysfunction using the NeuRow BCI-VR system. This is achieved through the acquisition of clinical scales, dynamics of EEG during the BCI treatment, and brain activation as measured by functional MRI (fMRI). NeuRow is an immersive VR environment for MI-BCI training that uses an embodied avatar representation of the patient arms and haptic feedback. The combination of MI-BCIs with VR can reinforce activation of motor brain areas, by promoting the illusion of physical movement and the sense of embodiment in VR (Slater, 2017), and hence further engaging specific neural networks and mobilizing the desired neuroplastic changes. Virtual representation of body parts paves the way to include action observation during treatment. Moreover, haptic feedback is added since a combination of feedback modalities could prove to be more effective in terms of motor-learning (Sigrist et al., 2013). Therefore, the target of this system is to be used by patients with low or no levels of motor control. With this integrated BCI-VR approach, severe cases of stroke survivors may be admitted to a VR rehabilitation program, complementing traditional treatment.

## Methodology

### Patient Profile

In this pilot study we recruited a 60 years old male patient with left hemiparesis following cerebral infarct in the right temporoparietal region 10 months before. The participant had corrected vision through eyewear, he had 4 years of schooling and his experience with computers was reported as low. Moreover, the patient was on a low dose of diazepam (5 mg at night to help sleep), dual antiplatelet therapy, anti-hypertensive drug and metformin. Hemiparesis was associated with reduced dexterity and fine motor function; however, sensitivity was not affected. Other sequelae of the stroke included hemiparetic gait and dysarthria. Moreover, a mild cognitive impairment was identified which did not interfere with his ability to perform the BCI-VR training. The patient had no other relevant comorbidities. Finally, the patient was undergoing physiotherapy and occupational therapy at the time of recruitment and had been treated with botulinum toxin infiltration 2 months before due to focal spasticity of the biceps brachii.

### Intervention Protocol

The patient underwent a 3-weeks intervention with NeuRow, resulting in 10 BCI sessions of a 15 min of exposure in VR training per session. Clinical scales, motor imagery capability assessment, and functional -together with structural- MRI data had been gathered in three time-periods: (1) before (serving as baseline), (2) shortly after the intervention and (3) one-month after the intervention (to assess the presence of long-term changes). Finally, electroencephalographic (EEG) data had been gathered during all sessions, resulting in more than 20 datasets of brain electrical activity.

The experimental protocol was designed in collaboration with the local healthcare system of Madeira, Portugal (SESARAM) and approved by the scientific and ethic committees of the Central Hospital of Funchal. Finally, written informed consent was obtained from the participant upon recruitment for participating to the study but also for the publication of the case report in accordance with the 1964 Declaration of Helsinki.

### Assessment Tools

A set of clinical scales were acquired including the following:

1. Montreal Cognitive Assessment (MoCA). MoCA is a cognitive screening tool, with a score range between 0 and 30 (a score greater than 26 is considered to be normal) validated also for the Portuguese population, (Nasreddine et al., 2005).
2. Modified Ashworth scale (MAS). MAS is a 6-point rating scale for measuring spasticity. The score range is 0, 1, 1+, 2, 3, and 4 (Ansari et al., 2008).
3. Fugl-Meyer Assessment (FMA). FMA is a stroke specific scale that assesses motor function, sensation, balance, joint range of motion and joint pain. The motor domain for the upper limb has a maximum score of 66 (Fugl-Meyer et al., 1975).
4. Stroke Impact Scale (SIS). SIS is a subjective scale of the perceived stroke impact and recovery as reported by the patient, validated for the Portuguese population. The score of each domain of the questionnaire ranges from 0 to 100 (Duncan et al., 1999).
5. Vividness of Movement Imagery Questionnaire (VMIQ2). VMIQ2 is an instrument that assess the capability of the participant to perform imagined movements from external perspective (EVI), internal perspective imagined movements (IVI) and finally, kinesthetic imagery (KI) (Roberts et al., 2008).

### NeuRow BCI-VR System

#### EEG Acquisition

**FIGURE 1:**
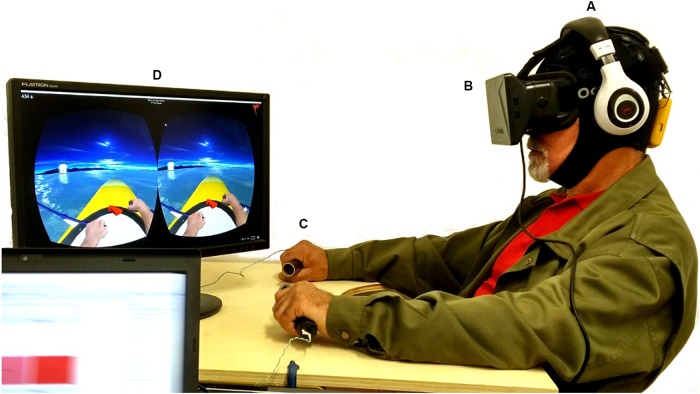
Experimental setup, including: **(A)** the wireless EEG system; **(B)** the Oculus HMD, together with headphones reproducing the ambient sound from the virtual environment; **(C)** the vibrotactile modules supported by a custom-made table-tray, similar to the wheelchair trays used for support; **(D)** the visual feedback with NeuRow game. A written informed consent was obtained for the publication of this image.

For EEG data acquisition, the Enobio 8 (Neuroelectrics, Barcelona, Spain) system was used. Enobio is a wearable wireless EEG sensor with 8 EEG channels for the recording and visualization of 24-bit EEG data at 500 Hz and a triaxial accelerometer. The spatial distribution of the electrodes followed the 10–20 system configuration (Klem et al., 1999) with the following electrodes over the somatosensory and motor areas: Frontal-Central (FC5, FC6), Central (C1, C2, C3, C4), and Central-Parietal (CP5, CP6) (Figure 1A). The EEG system was connected via Bluetooth to a dedicated desktop computer, responsible for the EEG signal processing and classification, streaming the data via UDP through the Reh@Panel (RehabNet Control Panel) for controlling the virtual environment. The Reh@Panel is a free tool that acts as a middleware between multiple interfaces and virtual environments (Vourvopoulos et al., 2013).

#### Head Mounted Display

For delivering the visual feedback to the user, the Oculus Rift DK1 HMD was used (Oculus VR, Irvine, CA, United States). The HMD is made of one 7″ 1280 × 800 60 Hz LCD display (640 × 800 resolution per eye), one aspheric acrylic lens per eye, 110^o^ Field of View (FOV), internal tracking through a gyroscope, accelerometer, and magnetometer, with a tracking frequency of 1000 Hz (Figure 1B).

#### Haptic Feedback

For delivering vibrotactile feedback, a custom module was used with out-of-the-box components including an Arduino Mega 2560 board and vibrating motors. The vibrating motors (10 mm diameter, 2.7 mm thick) performed at 11000 RPM at 5 V and were mounted inside cylindrical tubes -using 3D printed casing- which act as grasping objects for inducing the illusion of movement during the BCI task. In our setup, a pair of tubes with 12 cm (4.7 inches) of length and 3 cm (1.2 inches) diameter were used (Figure 1C).

#### VR Feedback

The BCI-VR task involved the use of NeuRow, a first-person BCI game (or neurogame). NeuRow uses a self-paced BCI paradigm and not a cue-based in order to increase the ecological validity of the training task. The actions are triggered whenever the user intends to move, like one would do in a real-life scenario. The BCI-VR task involves a boat rowing task through mental imagery with the goal of collecting as many flags as possible in a fixed amount of time. The auditory feedback involved two types of sounds. (1) background and ambient sounds of the water including rowing movement for increased realism and (2) event sounds for when the player was achieving a score by capturing a flag. NeuRow is a multiplatform virtual environment developed in Unity game engine (Unity Technologies, San Francisco, CA, United States). Finally, NeuRow was used under Windows OS, although it is optimized also for Android and Web browser by using the Reh@Panel (Vourvopoulos et al., 2016b).

The in-game interface includes time indication, score, navigational aids and first-person perspective of a virtual avatar representation of upper limbs rowing. NeuRow can be customized with different settings, depending on the experimental setup, BCI paradigm and running platform. NeuRow has two operating modes: (1) MI training and (2) online control. During training, the navigational arrow and the targets are removed to focus user’s attention only on the MI BCI-VR task. During online mode, the behavior of the boat can be changed by setting the heading speed, turn speed and cut-off angle of 45°. The cut-off angle is the allowed angle that the boat can be off-course with respect to the target flag before stopping. This serves as an additional safety feature to ensure that the user does not deviate from the target since the virtual environment is procedurally generated (Figure 1D).

### NeuRow BCI-VR Protocol

#### Training Session

**FIGURE 2:**
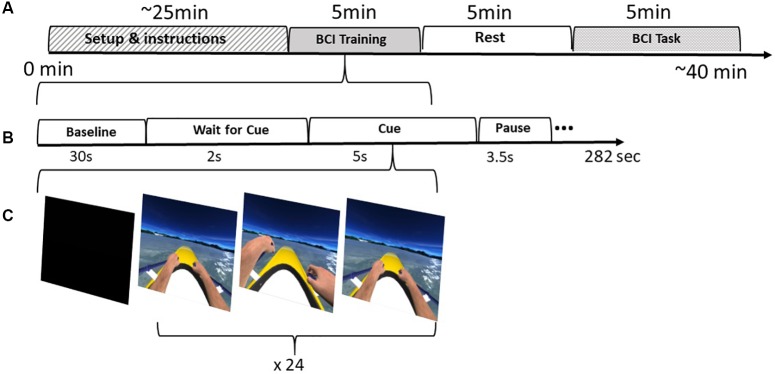
BCI Protocol: **(A)** Intervention stages including the setup, training, resting period and finally the BCI task. **(B)** The training stages. **(C)** Training feedback distributed in 24 epochs per class (left|right)

The first step of the training consisted on the acquisition of the raw EEG data to train a linear classifier to distinguish between Right and Left imagined hand movements. Throughout the training session, the user performed mental imagery of the corresponding hand rowing (left or right) at the same pace as the movement presented in VR. For each hand, the user is stimulated visually (VR action observation), auditorily, and haptically through the vibration on the corresponding hand. The training session was configured to acquire data in 24 blocks (epochs) per class (left or right hand imagery) in a randomized order (Figure 2).

#### Online EEG Data Processing

Following training, the acquired signals were processed with a bandpass filter (8–30 Hz), epoched into 4 s chunks following a stimulation event and used to compute a Common Spatial Patterns (CSP) filter. CSP was used in order to maximize the difference between the signals of the two classes (left vs right) for increased performance, and has become a standard tool in the use of MI-based BCIs (Lotte, 2014). Further, the training EEG data through the spatial filter feature vector were used to train a Linear Discriminant Analysis (LDA) classifier. LDA computed a vector which best discriminates between the two classes (left or right motor-imagery). Finally, the LDA output (hyperplane distance) was used as an input to the Adaptive Performance Engine (APE) (Ferreira et al., 2015). The APE module is composed a Bayesian Inference Layer (BIL) and a Finite State Machine (FSM) and it was added to smoothen binary decisions in a self-paced BCI scenario. BIL was computed as the likelihood of a specific LDA output belonging to each MI class with the following formula:

(1) $$\text{P}(\text{i}|\text{LDA}\cdot \text{output}=\frac{{\text{MI}}_{\text{i}}(\text{LDA}\cdot \text{output},{\;\mu }_{\text{j}},\sigma_{\text{j}})*{\text{P}}_{\text{i}}}{\sum_{\text{j}}{\text{MI}}_{\text{j}}(\text{LDA}\cdot \text{output},{\;\mu }_{\text{j}},\sigma_{\text{j}})}$$

where *P_i_* indicates the prior probability of action *i* (0.5 for left vs. right MI). μ and σ are updated at each iteration, taking into account all previous history of the user for the given *i* MI action. LDA output indicates the output value of the LDA classifier.

Next, the posterior probabilities of each class (left or right MI) were sent to the FSM. Each state in the FSM represents the class, but also the confidence level associated to them.

### EEG Offline Analysis

For the offline analysis, EEG signals were processed in MATLAB^®^ (The MathWorks, Natick, MA, United States) with the EEGLAB toolbox (Delorme and Makeig, 2004). After importing the data together with the channel info, a high-pass filter at 1 Hz was used to remove the “baseline drift” followed by line-noise and harmonics removal at 50 Hz. Furthermore, bad channels were rejected, and the data were subsequently re-referenced to average. Any potential missing channels had been interpolated to minimize a potential bias in the re-referencing stage through the pop_interp() function of EEGLAB which uses the spherical spline algorithm (Perrin et al., 1989). Next, an Independent Component Analysis (ICA) was performed for removing eye blinking, and movement artifacts (Makeig et al., 1996). For the independent components (IC) labeling, we performed manual artifact recognition by inspecting the different components both in the time and frequency domain but using also the ICLabel plugin from EEGLAB. The ICLabel plugin includes a trained classifier for EEG independent component which provides us with the probabilities that a component is being in any of the seven categories: brain; muscle; eye; heart; line noise; channel noise; other. The ICLabel classifier is trained by using crowd sourced data labeling or crowd labeling (Pion-Tonachini et al., 2017).

#### EEG Spectral Power

The Welch’s method for Power Spectral Density (PSD) of the power spectrum (Welch, 1967) was used for computing the average spectral power across the following frequency bands during the training task: Alpha (8–12 Hz), Beta (12–30 Hz), Theta (4–7 Hz), and Gamma (35–90 Hz). In addition, Alpha PSD was computed during resting-state before the trials.

#### Event-Related Desynchronization

In addition, the event-related synchronization/desynchronization (ERS/ERD) was extracted following the standard ERS/ERD method (Pfurtscheller and Aranibar, 1979) across the Mu band (8–12 Hz) and the Beta band (12–30 Hz). Both Mu and Beta power were extracted over C3 and C4 electrode locations. ERD was calculated by using the following formula:

(2) $$\begin{array}{c} {\text{ERD}}_{\text{C}3|\text{C}4}=(\text{PowerC}3|\text{C}4_{\text{MotorActivity}}-\text{PowerC}3|\text{C}4_{\text{Baseline}})\\ \;/\text{PowerC}3|\text{C}4_{\text{Baseline}}\times 100 \end{array}$$

With positive numbers indicating ERS and negative numbers indicating ERD.

Moreover, ERDS maps were extracted as a time/frequency representation of ERD/ERS between 8 and 30 Hz (Graimann et al., 2002). ERDS maps are also known as ERSP (event-related spectral perturbation) and act as a generalization of the ERS/ERD (Makeig, 1993).

#### Lateralization Index

Lateralization between hemispheres is generally assessed by a lateralization index (LI), commonly used to describe the asymmetry of neural activation intensity. In this study, LI was computed on the basis of the relative power values detected over C3 and C4 electrodes (Doyle et al., 2005). In order to quantify lateralization, the spectral power at electrodes, contralateral to the movement side, was subtracted from that at ipsilateral electrodes. Moreover, we have extracted the LI for both Mu ERD and Beta ERD during training in terms of relative power, with its sign indicating contralateral ERS (negative) or ERD (positive) dominance (Doyle et al., 2005). For example, if the contralateral value is smaller than the ipsilateral value, then LI value is positive, indicating contralaterally desynchronized status in the evoked ERD during MI. Finally, the LI was computed as the average of the right and left side differences using the following formula:

(3) $$\begin{array}{c} \text{LI}=[(\text{ERDC}3_{\text{Left movement}}-\text{ERDC}4_{\text{Left movement}}\\ +(\text{ERDC}4_{\text{Right movement}}-\text{ERDC}3_{\text{Right movement}})]/2 \end{array}$$

#### BCI Accuracy

For measuring the BCI performance during the training phase, the following formula was used for quantifying binary accuracy from the classifier:

(4) ACC=(TP+TN)/(TP+TN+FP+FN)

where: TP, true positive; FP, false positive; TN, true negative; FN, false negative.

### fMRI Acquisition and Analysis

#### Task

The patient was submitted to five fMRI consecutive runs at each of the three assessment periods, while executing the following tasks: finger-tapping execution with the non-affected hand (ME-Right), finger-tapping motor imagery with both left and right hands, separately (MI-Left and MI-Right), and motor imagery with NeuRow with both left and right hands, separately (MI-NeuRow-Left and MI-NeuRow-Right). Thus, a total of 3(periods) × 5(runs) = 15 fMRI runs were performed. For all conditions, each run consisted of 7 cycles alternating a 20 s block of baseline (fixation cross) followed by a 20 s block of task; yielding a total duration of each fMRI run of 5.33 min (Pimentel et al., 2013).

**FIGURE 3:**
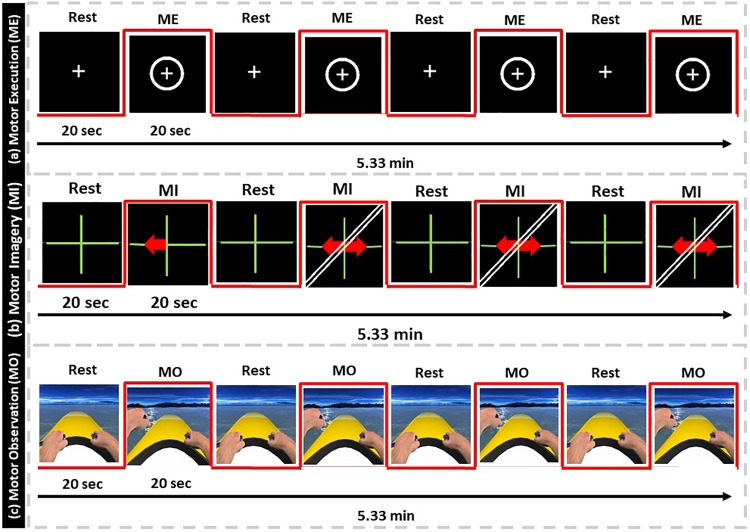
fMRI protocol. **(a)** Motor-Execution feedback, **(b)** Motor-Imagery feedback with directional arrows, **(c)** Motor-Observation feedback of NeuRow.

In the finger-tapping execution condition (ME), the patient was instructed to execute a sequential finger-tapping task (index-middle-ring-little-index-middle-ring-little) from a first-person perspective with his non-affected arm (Figure 3a). In the finger-tapping motor imagery condition (MI), the patient had to imagine the kinesthetic experience of the previous finger-tapping task for the left and right hand separately, based on the provided stimulus/instruction (Figure 3b). Each trial started with a fixation cross, followed by a red arrow pointing to the left or right, indicating the beginning of a movement execution/imagination period, known as the standard Graz Motor Imagery protocol. In the last condition, the motor observation (MO), patient had to observe and imagine the kinesthetic experience of the rowing task from NeuRow from the first-person perspective for the left and right hand separately, based on the provided stimulus (Figure 3c). The fixation cross (baseline) from the motor imagery condition was replaced by NeuRow feedback in idle (floating without rowing); then, the corresponding movement was initiated for left or right rowing.

For the fMRI data acquisition, only visual and auditory feedback was delivered. The visual feedback was delivered through specialized MR-compatible fiber-optic goggles at a resolution of 640 × 480 pixels, synchronized with the console computer. The sound was delivered through MR compatible pneumatic headphones, but no haptic feedback was delivered during the fMRI session due to the lack of MR compatible equipment.

#### Image Acquisition

Imaging was performed on a 3T GE Signa HDxt MRI scanner (General Electrics Healthcare, Little Chalfont, United Kingdom) using 12-channel receive head coil. Functional images were acquired using a 2D multi-slice gradient-echo echo-planar imaging (EPI) sequence, with TR/TE = 2500/30 ms, flip angle = 90°c, and FOV = 224 × 224 mm^2^, from 36 contiguous axial slices with interleaved acquisition, and 3.5 × 3.5 × 3.5 mm^3^ voxel size (0.75 mm slice gap), yielding whole-brain coverage. Whole-brain, structural images were acquired using a T_1_-weighted 3D fast spoiled gradient-echo (FSPGR) sequence, with 1.0 × 1.0 × 0.6 mm^3^ voxel size.

#### Image Pre-processing

The following pre-processing steps were applied to the fMRI data recorded at all periods and runs prior to subsequent analyses. The first three volumes were discarded to allow the signal to reach the steady-state, and non-brain tissue was removed using FSL’s tool BET (Smith, 2002). Subsequently, slice timing and motion correction were performed using FSL’s tool MCFLIRT (Jenkinson et al., 2002), followed by high-pass temporal filtering with a cut-off period of 100 s and spatial smoothing using a Gaussian kernel with full width at half-maximum (FWHM) of 5 mm.

Because of the contamination by head motion as well as other physiological and instrumental artifacts, the pre-processed fMRI data were further submitted to an independent component analysis (ICA) cleaning procedure. Specifically, the probabilistic spatial ICA decomposition was applied, as implemented in the FSL’s tool MELODIC (Beckmann and Smith, 2004), with the default parameters, including the MELODIC’s automatic dimensionality estimation, as recommended in Salimi-Khorshidi et al. (2014). The purpose of ICA in this case was to separate neuronal from non-neuronal fMRI spatially independent components (ICs). The automatic classification of ICs was performed using FSL’s tool FIX (Salimi-Khorshidi et al., 2014). This tool extracts a large number of temporal and spatial features to be fed into a core classifier previously trained using hand-labeled components. The standard training weights were used, as the image acquisition parameters for our fMRI data. The non-neuronal related ICs were then automatically classified by FIX, and subsequently removed from the back-reconstruction step of the fMRI data, yielding cleaned fMRI data.

#### Statistical Analysis

For the purpose of mapping the brain areas involved in each fMRI run (left finger-tapping, left and right motor imagery, and left and right motor imagery with NeuRow), a general linear model (GLM) analysis of the pre-processed and ICA-based cleaned fMRI data was conducted. For each of the five fMRI runs, the explanatory variable of interest of the GLM was defined as a boxcar function, with 0’s during the baseline periods (fixation cross or boat floating without rowing) and 1’s during the task periods, and subsequently convolving it with a canonical double-gamma hemodynamic response function (Friston et al., 1995). A GLM containing the explanatory variable of interest, as well as the six motion parameters (rotation and translation of the head across the three main axes) estimated by MCFLIRT as confounding explanatory variables, was fit to the pre-processed data using FSL’s improved linear model (FILM). A *t*-test was performed on the parameter estimate for the explanatory variable of interest in each voxel and converted to Z-score. The resulting statistical parametric maps were subjected to cluster thresholding (voxel *Z* > 2.3, cluster *p* < 0.05) in order to yield the brain activation maps associated with each task (Woolrich et al., 2001).

The quantification of brain activation with each task was performed for each cerebral hemisphere separately and was based on the number of voxels exhibiting significant activation (voxels surviving the statistical thresholding; *N_vox_*), and the maximum Z-score (*Z_max_*). While the former approximately reflects the extent of brain activation, the latter approximately reflects its intensity.

## Results

Here we present the results from the clinical scales, the MI capability assessment and the fMRI data, obtained in the three time-periods (pre; post; follow-up). In addition, we present results of the BCI performance over all sessions and the extracted EEG data, compared between the first and the last session.

### Clinical Scales

In terms of motor domain as extracted by the FMA scale for the upper extremity (FMA-UE), the patient showed an improvement of 9 points at the end of the intervention (pre: 31, post: 40), followed by an improvement of 4 points (follow-up: 44) after 1 month (Table 1). This improvement is within the estimated clinically important difference (CID) scores, ranging between 4.25 and 7.25 points (Page et al., 2012). Moreover, by comparing the CID with the mean scores of prior BCI studies (*M* = 7.5, *SD* = 3.6) together with control groups (*M* = 4, *SD* = 2.2) (Mihara et al., 2013; Ramos-Murguialday et al., 2013; Ang et al., 2014, 2015; Li et al., 2014; Pichiorri et al., 2015; Frolov et al., 2016; Kim et al., 2016; Leeb et al., 2016), we are able to identify a big improvement of the patient in relation to both groups (Supplementary Figure 3).

**Table 1:** Clinical scales: Fugl-Meyer upper limb scale (FMA), Montreal Cognitive Assessment (MoCA) and Modified Ashworth Scale (MAS).

	Pre	Post	Follow-up
FMA	31	40	44
MoCA	20	21	18
MAS	1+	2	1+

Concerning spasticity, muscle tonus was increased but did not interfere with range of motion. SIS showed a conspicuous increase in the strength domain, however, the overall subjective assessment of the patient over his recovery remained constant (Table 2).

**Table 2:** Stroke **i**mpact **s**cale SIS subscales.

	Pre	Post	Follow-up
Strength	50	87.5	87.5
Hand function	100	95	95
Mobility	100	100	100
Memory	100	100	100
ADL and IADL	95	97.5	97.5
Communication	100	100	100
Emotion	97.2	94.4	94.4
Handicap	100	87.5	87.5
Physical domain	86.3	95	95
Stroke recovery	70	70	70

### Motor-Imagery Capability

#### VMIQ Pre-Post-follow

The capability for vivid MI, was assessed through 3 sub-scales of VMIQ-2, external visual imagery (EVI), internal visual imagery (IVI) and finally kinesthetic imagery (KI) (Table 3). For EVI an increased visual capability was reported after the intervention while maintained in the follow-up (pre:19, post: 47, follow-up: 47). Further, the IVI had a small increase post-intervention but returned to the same level after 1 month (pre:47, post: 48, follow-up: 47). Regarding KI, while it remained stable at the pre-post assessment, a lower level was reported at the follow-up (pre:47, post: 47, follow-up: 39) accompanied by a slight decrease in KI vividness. This may be explained by the fact that during the one-month period after intervention, the patient did not undergo any BCI training nor exercises involving kinesthetic imagery. Nonetheless, there was still a strong use of external and internal imagery.

**Table 3:** VMIQ-2 subscales of External Visual Imagery (EVI), Internal Visual Imagery (IVI) and Kinesthetic Imagery (KI).

	Pre	Post	Follow-up
EVI	19	47	47
IVI	47	48	47
KI	43	44	39

#### Comparison With Healthy Participants

**FIGURE 4:**
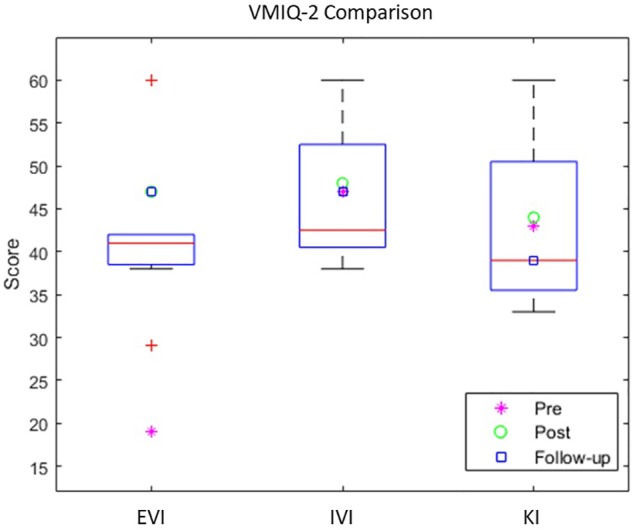
VMIQ-2 subscales comparison of Pre, Post and Follow-up scores with healthy data

Comparing the MI capability data of the VMIQ-2 questionnaire with a group of healthy participants (*N* = 8) that underwent the same BCI protocol from a previous study (Vourvopoulos et al., 2016b), we can estimate a “healthy” range for motor-imagery capability of healthy population as a reference (Figure 4). Concerning the difference in EVI comparing pre-post assessments of our patient, we can observe a pronounced leap after the BCI-VR intervention, overpassing the average score of the healthy group (Figure 4). In contrast, the IVI and KI scores (that showed to be stable), are within the healthy range of the reported motor-imagery capability.

### BCI Performance

#### Classifier Performance

**FIGURE 5:**
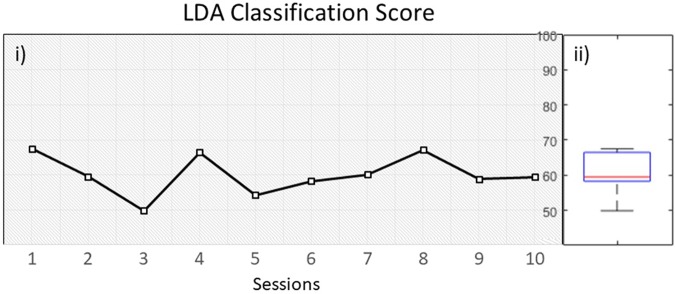
**(i)** LDA classification performance over time within 10 sessions, **(ii)** distribution of performance.

The overall classification performance during the 10 training sessions, was kept relatively low (*M* = 60, *SD* = 5.7) (Figure 5). Since we trained out classifier with features originating from both beta and mu bands, we wanted to investigate if there is a dominant frequency that could help achieving increased classification performance. A pair-samples *t*-test over C3 [*t*(9) = −0.9968, *p* = 0.34] and C4 [*t*(9) = 1.0878, *p* = 0.3049] during MI of the paretic arm, revealed non-significant differences between beta and mu ERD (Supplementary Figure 1).

Next, we compared the classification score across all sessions with the mean of two groups of healthy users. First with a group of participants (*N* = 8) that underwent the same BCI protocol with NeuRow (Vourvopoulos et al., 2016b) from a previous study (VR group) and secondly with a group of a study (*N* = 12) (Vourvopoulos et al., 2016a) that used the same feature extraction method (band power with CSP) and classification (LDA) for two-classes (left/right hand) MI data but not with VR feedback (non-VR group).

**FIGURE 6:**
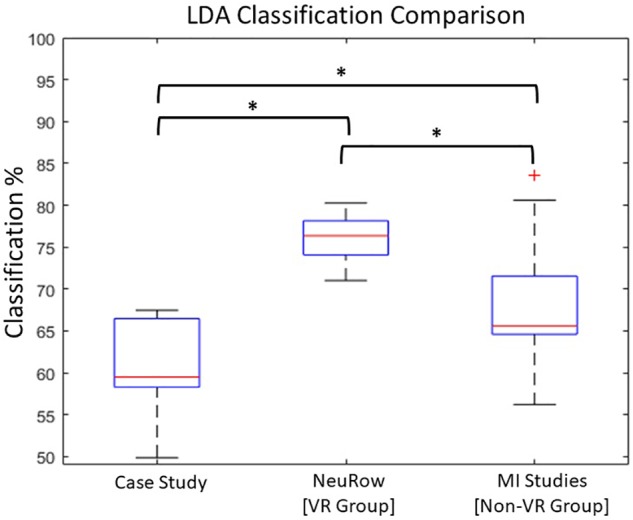
LDA Comparison with healthy. Statistically significant differences between Case-study, VR and non-VR groups has been observed (^∗^*p* < 0.05).

An independent sample *t*-test revealed significant differences between the patient LDA score (*M* = 60, *SD* = 5.7) with the VR group (*M* = 76, *SD* = 3), *t*(16) = −7.121. *p* < 0.001, but also with the non-VR group (*M* = 68, *SD* = 7.7), *t*(20) = −2.730, *p* < 0.05. Moreover, a significant difference was also found between the healthy VR and non-VR groups, *t*(18) = −2.720. *p* < 0.05 (Figure 6). Despite data showing improved performance with VR, our patient performed close to healthy in non-VR settings.

### EEG

Resting state Alpha band modulation is found to be related with cognitive and motor performance in stroke patients (Dubovik et al., 2012, 2013). We therefore analyzed the resting state Alpha rhythm pre and -post intervention (Supplementary Figure 2). Our results showed an increase in modulation of Alpha from Pre (*M* = 0.44, *SD* = 0.05) to Post (*M* = 0.62, *SD* = 0.24) although a paired-samples *t*-test yielded no significant differences (*p* = 0.20).

**FIGURE 7:**
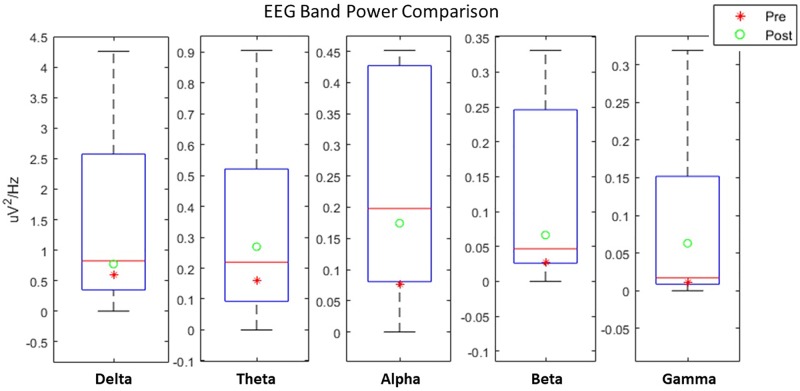
EEG spectral power comparison Pre-Post the intervention with healthy user data.

Furthermore, by comparing the evoked EEG activity during training with a prior study with NeuRow (Vourvopoulos et al., 2016b), we can observe a consistent trend between the first and last session. EEG power from healthy participants using the same experimental apparatus, can be used as a proxy for “healthy” EEG modulation boundaries. For all EEG bands, we found that the EEG power on the first session (Pre) is in the lower quartile (Q1) of the distribution while on the last session the EEG power always increases, closer approximating the Median of the healthy distribution inside the Interquartile Range (IQR). Current results indicate a convergence toward the healthy group EEG power (Figure 7).

**FIGURE 8:**
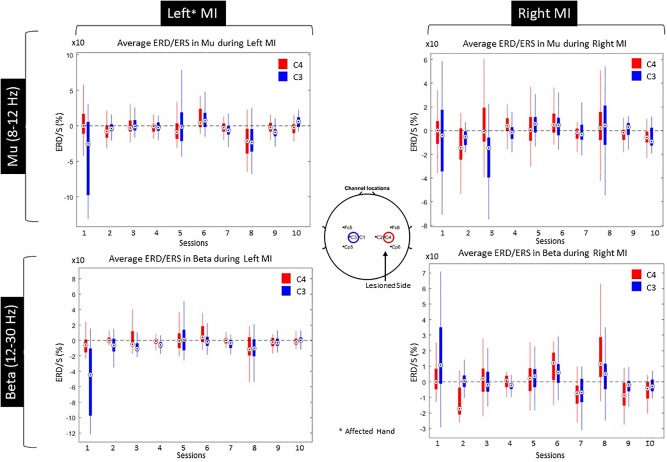
ERS/ERD during Left and Right MI for both Mu and Beta bands.

Since both movement and imagery are associated with Mu and Beta rhythm desynchronization (McFarland et al., 2000), by measuring ERD during the patients training, we anticipated stronger ERD (in terms of higher negative percentage compared to baseline) at the end of the intervention. Nonetheless, the evoked ERD had small power in both Mu and Beta bands (Figure 8).

**FIGURE 9:**
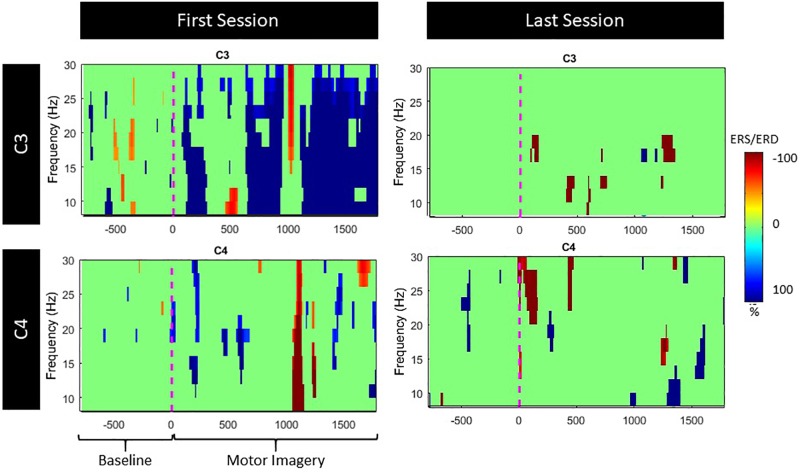
ERS/ERD activation maps during left (lesioned) hand motor-imagery. Significant ERD is illustrated with blue.

The ERD activation maps were extracted as a time/frequency representation of the first and last session during MI from the affected hand (left). Maps illustrate a clear desynchronization in the band 8–30 Hz -compared to baseline- from the contralesional electrode (C3) but not the ipsilesional side (C4). Nonetheless, in the last session, ERD is decreased but it is also more balanced (Figure 9).

**FIGURE 10:**
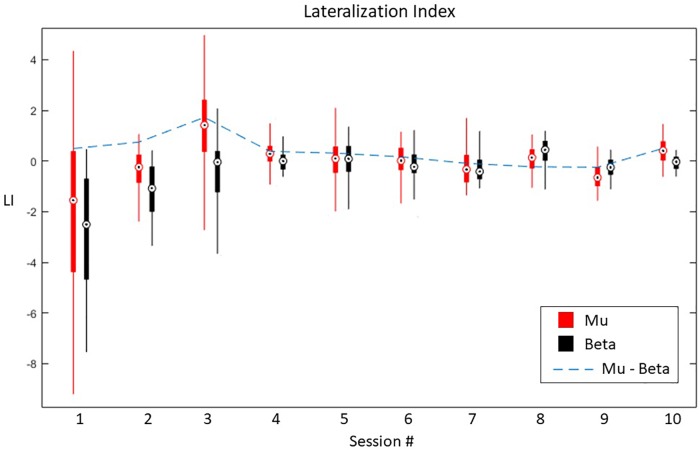
Lateralization index for Mu and Beta bands across all sessions.

In terms of laterality, LI shows an ipsilateral ERD dominance, evolving toward a contralateral ERD over time, balancing for both Mu and Beta bands (Figure 10). Furthermore, a paired-samples *t*-test revealed significant differences between the first and the last session in terms of LI ERD. Specifically, Beta band had increased ipsilateral ERD dominance in the first session (*M* = −2.82, *SD* = 2.3), balancing for both hemispheres in the last session (*M* = −0.6, *SD* = 0.3), *t*(199) = −16.921, *p* < 0.001. Similar trend for Mu band between the first (*M* = −1.8, *SD* = 3.1) and the last session (*M* = 0.41, *SD* = 0.5), *t*(199) = −9.832, *p* < 0.001.

### fMRI

**FIGURE 11:**
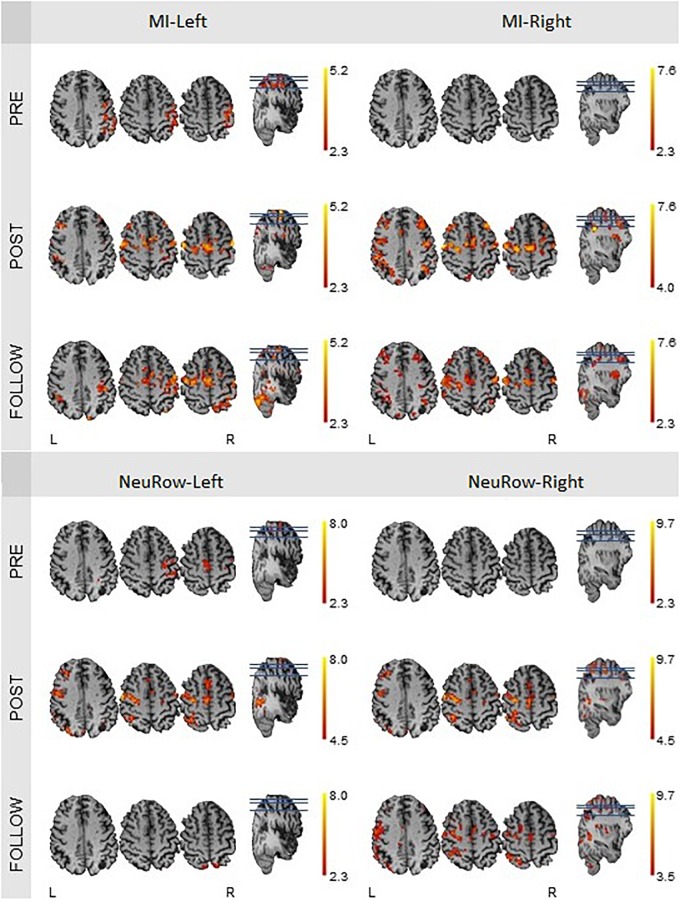
fMRI activation maps of the motor imagery condition for the left and right hand, at the pre-intervention, post-intervention and follow-up recording sessions. The *N*_vox_ and *Z*_max_ values are associated with the hemisphere contralateral to the hand. All maps have a threshold at *Z* > 2.3, except for the one of the motor-imagery of the right hand at the post-intervention (*Z* > 4.0), because of the substantial higher *Z*_max_.

The quantification of brain activation (*N*_vox_ and *Z*_max_ values) for the finger-tapping motor imagery conditions without NeuRow (MI-Left and MI-Right) and with NeuRow (MI-NeuRow-Left and MI-NeuRow Right) is depicted in Table 4; in Figure 11, the brain activation maps for these conditions are presented, separately for the left and right hand.

**Table 4:** Quantification of brain activation in terms of number of voxels exhibiting significant activation (N_vox_) maximum *Z*-score (*Z*_max_) for the finger-tapping motor imagery conditions (MI-Left and MI-Right) and NeuRow condition, displaying the BCI-VR feedback (NeuRow-Left and NeuRow-Right).

Period	MI-Left				MI-Right				NeuRow-Left				NeuRow-Right			
	*Left Hemisphere (lesion)*		*Right Hemisphere*		*Left Hemisphere (lesion)*		*Right Hemisphere*		*Left Hemisphere (lesion)*		*Right Hemisphere*		*Left Hemisphere (lesion)*		*Right Hemisphere*	
	N_vox_	*Z*_max_	N_vox_	*Z*_max_	N_vox_	*Z*_max_	N_vox_	*Z*_max_	N_vox_	*Z*_max_	N_vox_	*Z*_max_	N_vox_	*Z*_max_	N_vox_	*Z*_max_
PRE	0	0.0	101	3.5	0	0.0	0	0.0	44	3.8	40	3.9	0	0.0	0	0.0
POST	277	4.2	260	5.2	377	7.6	255	6.8	89	6.5	117	7.9	356	8.4	124	8.8
FOLLOW	286	4.9	455	4.7	526	5.4	493	5.6	4	3.2	3	2.8	442	6.9	101	6.4

We found that the intensity of brain activation during MI and MI-NeuRow relative to baseline (quantified by Z_max_) increased substantially from the pre-intervention to the post-intervention study and was sustained at the follow-up study (except for the MI-NeuRow-Left, which decreased at the follow-up study). Importantly, by adding the NeuRow to the MI tasks, an increase in Z_max_ was observed at all study periods, thus supporting the relevance of NeuRow to improve the capability of the patient to perform the MI task. Moreover, an increase in the extent of brain activation (quantified by N_vox_) was also observed when comparing the pre-intervention with the post-intervention and follow-up studies for all conditions. Interestingly, the lesioned (left) hemisphere was not recruited during either left or right MI conditions, nor the right MI-NeuRow condition, at the pre-intervention study (*N*_vox_ = 0); and this was reversed with the intervention (MI-Left: *N*_vox_ = 277; MI-Right: *N*_vox_ = 377; MI-NeuRow-Right: *N*_vox_ = 356) and sustained at the follow-up study (MI-Left: *N*_vox_ = 286; MI-Right: *N*_vox_ = 526; MI-NeuRow-Right: *N*_vox_ = 442). These activation maps evidence the recruitment of brain regions known to be associated with motor activation and imagery (primary motor cortex and supplementary motor area), including those in the lesioned hemisphere. In contrast with the intensity of brain activation, it was not found differences in the extent of brain activation when comparing MI and MI-NeuRow at their respective study periods.

## Discussion

Our results, with an initial case study show clear improvements and recovery regarding motor function in terms of clinical scales, self-reported scales, electrophysiological data and brain imaging data.

In terms of clinical scales, FMA has shown a stable increase in motor functioning followed throughout all assessments. This so-called carryover effect is known to involve specific mechanisms of action based on movement prediction and sense of agency/body ownership. Moreover, this effect influences the ability of a patient to plan the movement and to perceive the stimulation as a part of his/her own control loop (Gandolla et al., 2016). That could indicate effective motor learning partially evoked by immersive VR through NeuRow. We therefore hypothesize that a multimodal immersive BCI-VR training could have enhanced the carryover effect of the rehabilitation process that could eventually be reflected in terms of improved motor function. This is in line with recent work in BCI’s for rehabilitation in chronic stroke patients, showing the possibility of long-lasting improvements in motor function (Ramos-Murguialday et al., 2019).

Moreover, despite the low MoCA score (as most stroke patients may show some level of cognitive impairment) – together with the low computer knowledge and only 4 years of schooling – the patient was able to learn and use motor imagery as a way to interact with the BCI-VR system.

In terms of the perceived impact of stroke through the SIS questionnaire, the results were overall ambiguous. Part of the variation could be due to internal factors of the patient such as his mood and optimism or level of frustration upon answering the questionnaire. Many items of the questionnaire require other functions such as posture, balance, proprioception and lower limb function, which were not targeted in the BCI intervention. The improvement in perceived muscle strength is of interest because of its direct correlation with upper limb motor improved, particularly in context with improved FMA scores of the upper limb.

Further, increased MI ability as reported by VMIQ-2, but also as captured by the EEG data through the Alpha and Beta bands, seem to manifest the potential of motor recovery. Hence, current methodology for motor-imagery training may provide a valuable tool to access the motor network and improve outcome after stroke. This is also in-line with prior research findings illustrating better functional outcome in the BCI group, including a significantly higher probability of achieving a clinically relevant increase in the FMA score (Pichiorri et al., 2015). Additionally, the comparison with healthy data, reveals a convergence toward the healthy motor-imagery dataset in all domains (external, interval and kinesthetic), while also maintaining a high score in follow-up.

In terms of EEG, by comparing resting state Alpha rhythm, between the first and the last session, we observed an increase in power. Moreover, using as a point of reference healthy data -undergoing the same BCI training-, we can see that after the intervention, EEG data are closer to the distribution of the healthy participants. Since MI roughly involves (to a large extent) the same cortical areas that are activated during actual motor preparation and execution (Jeannerod and Frak, 1999), this increase is likely to be indicative of motor recovery.

Furthermore, the evoked ERD during MI training did not have the anticipated power in either Mu or Beta bands. This probably explains the low LDA classification score, showing that low performance is a direct result of the evoked EEG activation and not due to classifier configuration.

Overall, BCI performance quantified in terms of LDA classification score was stable throughout all sessions. In addition, compared with two healthy groups (VR and non-VR), we can see again that VR can result into better classification scores compared with standard training (Vourvopoulos and Bermúdez, 2016a; Vourvopoulos et al., 2016b), although our patient showed a lower performance. This can highlight once more the importance of the VR feedback and the role of agency in BCI performance.

In terms of LI, previous studies have showed that movement-related neural activity is lateralized, particularly those using fMRI (Babiloni et al., 2003). Moreover, brain activation symmetry is modified after stroke due to the resulting one-sided lesion. Analysis of the lateralization and hemispheric asymmetries of neural activity might provide a valuable neurophysiological parameter in the prognosis and follow-up of patients (Cicinelli et al., 2003).

Finally, the analysis of the fMRI data showed evidence of plastic changes including recruitment in the primary motor cortex and supplementary motor area, brain regions known to be associated with motor activation and imagery, including those in the lesioned hemisphere.

Therefore, a tailored BCI-VR training paradigm could help preventing maladaptive plasticity -avoiding compensatory movements- while helping to develop normal movement patterns.

## Conclusion

With this case study, we have been able to test our proposed BCI-VR paradigm, acquiring information from various sources. Clinical scales illustrated improvements in motor function, electrophysiological data showed an increase in brain activation -similar to healthy subjects and brain-imaging data have showed the effect of MI training and VR feedback, promoting plastic changes in the targeted areas of the brain. Our findings extend prior research that showed the efficacy of BCIs using MI for motor rehabilitation (Silvoni et al., 2011; Pichiorri et al., 2015; Ramos-Murguialday et al., 2019). However, the majority of previous studies have not addressed the effect of self-paced and ecologically valid scenarios through VR feedback. These results suggest that this approach could be useful with chronic stroke patients with reduced upper limb motor function. As this is a case study, additional research is needed to explore this hypothesis including combined brain data with electrophysiological information during training. This will allow us to develop a tailored BCI-VR training paradigm that could help preventing maladaptive plasticity (e.g., by avoiding compensatory movements) and help to develop normal movement patterns. Finally, this will enable us also to identify the specific benefits of brain-controlled VR training environments for neurorehabilitation.

## Limitations

Although this study collected and explored 480 trials (240 per class) of post-stroke EEG signals, along with pre- and post-intervention fMRI, and clinical datasets, it is limited by its sample size. Moreover, in the absence of a control group, it is not clear to what extent conventional treatment lead to the improvements seen. Our findings, therefore, are preliminary, have limited statistical power, and should be interpreted with caution. In addition, variability in EEG band power is rather high, thus conclusion based on comparing the values for the first and the last sessions are exploratory and not confirmative.

## Data Availability

The datasets generated for this study are available on request to the corresponding author.

## Ethics Statement

The experimental protocol was designed in collaboration with the local healthcare system of Madeira, Portugal (SESARAM) and approved by the scientific and ethic committees of the Central Hospital of Funchal. Finally, an informed consent was obtained from the participant upon recruitment in accordance with the 1964 Declaration of Helsinki.

## Author Contributions

AV and SBiB defined and designed the research study. AV participated in the development of the software. CJ and J-CF conducted the clinical assessments. AV, CJ, and J-CF collected the data. AV analyzed the EEG data. CJ analyzed the clinical scales. RA and PF analyzed the fMRI data. All authors interpreted the data. SBiB supervised the study. All authors revised and approved the final version of the manuscript.

## Conflict of Interest Statement

The authors declare that the research was conducted in the absence of any commercial or financial relationships that could be construed as a potential conflict of interest.
