# Supplementary material for: Efficacy and Brain Imaging Correlates of an Immersive Motor Imagery BCI-Driven VR System for Upper Limb Motor Rehabilitation: A Clinical Case Report
Source: Front Hum Neurosci. 2019 Jul 11;13:244. doi: 10.3389/fnhum.2019.00244 (PMC6637378; doi:10.3389/fnhum.2019.00244)
Supplement: Supplementary file 1 [file Table_1.DOCX]

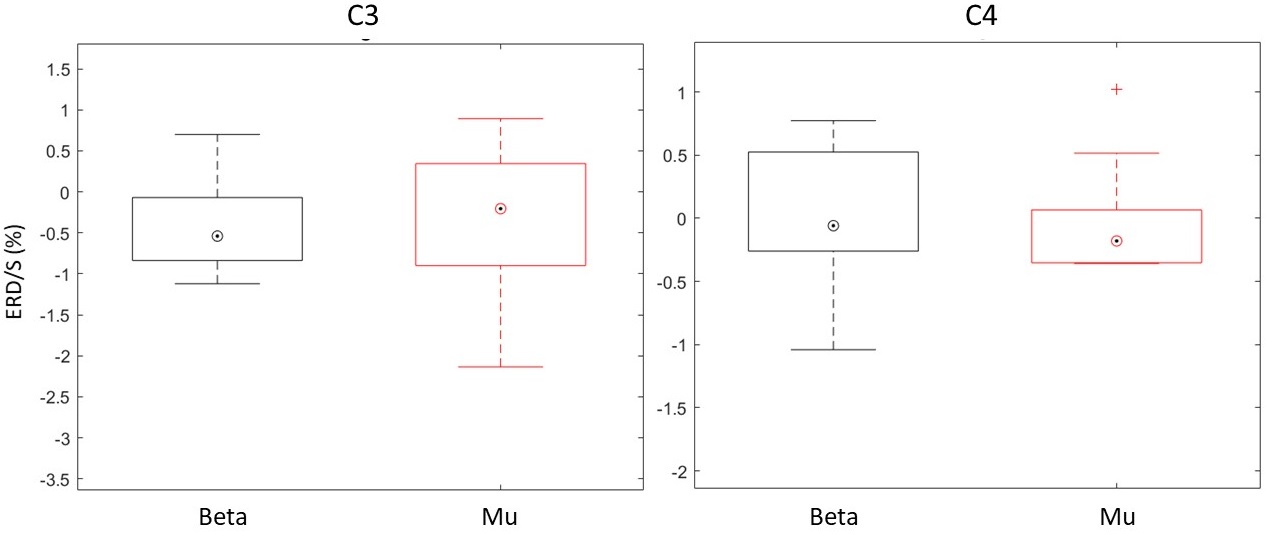


**Supplementary Figure 1.** Beta and mu ERD during MI of the paretic arm over C3 (left) and C4 (right)


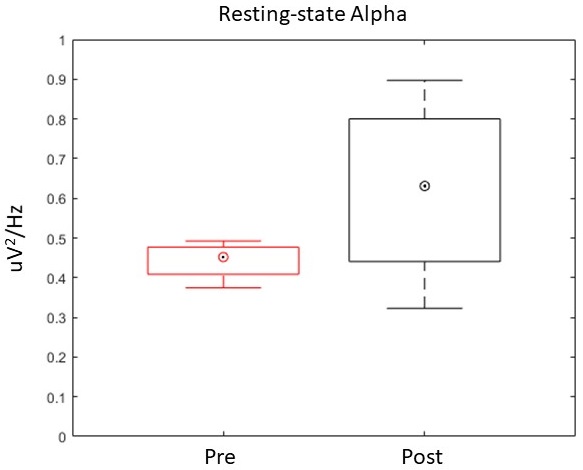


**Supplementary Figure 2.** Resting state Alpha Pre-Post intervention.


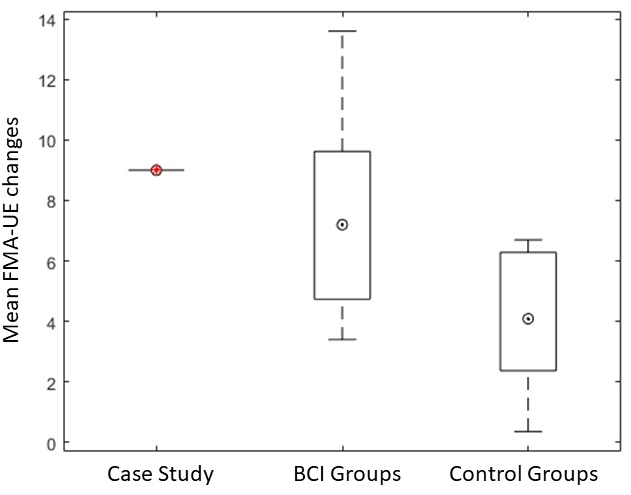


**Supplementary Figure 3.** Comparison of mean FMA‐UE changes in terms of clinically important difference (CID) between the case study patient and scores from prior studies with BCI and control groups (Leeb et al. 2016; Ramos-Murguialday et al. 2013; Pichiorri et al. 2015; Mihara et al. 2013; Li et al. 2014; Kim, Kim, and Lee 2016; Ang et al. 2015, 2014; Frolov et al. 2016). Adapted from (Cervera et al. 2018).
